# Cohabitation With Atlantic Salmon (*Salmo salar*) Affects Brain Neuromodulators But Not Welfare Indicators in Lumpfish (*Cyclopterus lumpus*)

**DOI:** 10.3389/fphys.2022.781519

**Published:** 2022-03-02

**Authors:** Fredrik R. Staven, Manuel Gesto, Martin H. Iversen, Per Andersen, Deepti M. Patel, Jarle T. Nordeide, Torstein Kristensen

**Affiliations:** ^1^Faculty of Biosciences and Aquaculture, Nord University, Bodø, Norway; ^2^Department of Research and Development, Aqua Kompetanse AS, Flatanger, Norway; ^3^Section for Aquaculture, Technical University of Denmark, Hirtshals, Denmark

**Keywords:** lumpfish, Atlantic salmon, cleaner fish, aquaculture, welfare, neuromodulators, habituation

## Abstract

Lumpfish are utilized to combat ectoparasitic epidemics in salmon farming. Research gaps on both cleaning behavior and client preferences in a natural environment, emphasizes the need to investigate the physiological impacts on lumpfish during cohabitation with piscivorous Atlantic salmon. Lumpfish (39.9 g, S.D ± 8.98) were arranged in duplicate tanks (*n* = 40 per treatment) and exposed to Live Atlantic salmon (245.7 g, S.D ± 25.05), salmon Olfaction or lifelike salmon Models for 6 weeks. Growth and health scores were measured every second week. In addition, the final sampling included measurements of neuromodulators, body color, and plasma cortisol. A stimulation and suppression test of the hypothalamic-pituitary-interrenal (HPI) axis was used for chronic stress assessment. Results showed that growth, health scores, and body color remained unaffected by treatments. Significant reductions in levels of brain dopamine and norepinephrine were observed in Live compared to Control. Plasma cortisol was low in all treatments, while the stimulation and suppression test of the HPI axis revealed no indications of chronic stress. This study presents novel findings on the impact on neuromodulators from Atlantic salmon interaction in the lumpfish brain. We argue that the downregulation of dopamine and norepinephrine indicate plastic adjustments to cohabitation with no negative effect on the species. This is in accordance with no observed deviations in welfare measurements, including growth, health scores, body color, and stress. We conclude that exposure to salmon or salmon cues did not impact the welfare of the species in our laboratory setup, and that neuromodulators are affected by heterospecific interaction.

## Introduction

The presence of predators induces multiple behavioral and physiological responses in prey animals. Among teleost fish, such responses involve freezing, fight, or flight; each strategy or a mix of strategies aimed at enhancing the likelihood for survival (Eaton et al., 1977; Clinchy et al., 2013; Rupia et al., 2016). Recognition of potential predators can be inherited and entails adapted systems for both sensory detection and defensive responses to external threats (Brown and Godin, 1999; Berejikian et al., 2003; Brown, 2003). Both olfaction and vision are essential sensory detection systems in aquatic prey fish, due to variable turbidity and light conditions in the environment (Chivers and Smith, 1998; Ferrari et al., 2010). For a prey fish, the time spent on high alert is time lost on fitness-related activities such as foraging and reproduction (Schreck, 2010). As a counter mechanism, habituation and threat-sensitive predator avoidance occur through plastic cognitive development when physiological and behavioral mechanisms are altered to no longer respond to a repeated non-specific risk (Brown et al., 2006; Rankin et al., 2009). A textbook example of adaptive plasticity to predators is observed among cleaner fish. Cleaner fish are specialized in the removal of ectoparasites from other fish, here called clients. Natural selection on habituation to client interactions have earned cleaner fish unique access to both parasite food items and nipping of nutrient rich skin mucus, delicately balancing mutualism over parasitism (Grutter, 1999; Bshary, 2001; Gingins et al., 2017). The behavioral approach sometimes involves cleaner fish signaling to the client fish, who respond with a non-aggressive behavior before parasites are removed (Grutter, 2004). In a cleaner fish–client fish interaction, the importance of habituation might thus seem apparent. To access ectoparasites, cleaners can approach much larger predator clients and perform behaviors most perilous, such as entering the clients mouth cavity in search of food items (Brown and Godin, 1999; Bshary and Würth, 2001).

In Atlantic salmon (*Salmo salar*) aquaculture, certain wrasse species (Labridae) and lumpfish (*Cyclopterus lumpus*) are utilized as cleaner fish to combat ectoparasites (Bjordal, 1990; Imsland et al., 2014a; Blanco Gonzalez and de Boer, 2017). Failing to control epidemics of the sea lice copepod *Lepheophteirus salmonis* have both economic and environmental consequences (Costello, 2009; Torrissen et al., 2013; Øverli et al., 2014). Today, one of the most common species of cleaner fish used in Atlantic salmon farming is lumpfish (Powell et al., 2017). Studies in commercial large-scale sea cages with Atlantic salmon, revealed significant reductions in sea lice numbers when juvenile lumpfish were added, where 13–36% of the lumpfish stock had sea lice in their stomach (Eliasen et al., 2018; Imsland et al., 2018a). Why sea lice grazing on Atlantic salmon is not homogeneous among all lumpfish individuals is uncertain, but variation between family strains have been observed (Imsland et al., 2016a). Ontogeny is also relevant for sea lice consumption, where small individuals (20–50 g) have been observed with a higher total count of sea lice in their stomach (Imsland et al., 2016b; Eliasen et al., 2018). With individual variation in behavior, it is thus relevant to assess physiological mechanisms involved in social situations.

The main challenges of using lumpfish in aquaculture are high mortality rates and the maintenance of good welfare during the commercial sea phase together with Atlantic salmon. It is necessary to investigate each part of the puzzle related to increased mortality in the myriad of stressors capable of impeding the resting state of lumpfish. One such stressor is social interaction with Atlantic salmon and the ability to habituate to the presence of a larger, potentially predatory, species. Whether lumpfish has been documented to be predated upon by Atlantic salmon in a commercial fish farm is uncertain. Nonetheless, Imsland et al. (2014b) observed no antagonistic behavior during cohabitation with the two species. While cleaner-client behavioral interactions have been documented for decades in aquaculture (Bjordal, 1990), including almost 10 years of research on lumpfish (Imsland et al., 2014a), knowledge gaps still exist on the underlying mechanisms that regulate sea lice grazing. Recently, the neurobiological, and neuro-molecular mechanisms involved in interspecific interaction have been investigated in other cleaner fish species (Soares, 2017; Ramírez-Calero et al., 2021). These novel approaches have accentuated the role of neuromodulators in social contexts (Soares et al., 2012, 2017; Paula et al., 2015; de Abreu et al., 2018, 2020). Neurotransmitters of relevance are dopamine, which influence Bluestreak wrasse (*Labroides dimidiatus*) learning capacity in cooperative investment, and time spent in physical contact with clients (Messias et al., 2016a, b; Soares et al., 2017). Serotonin has multiple roles including regulation of hypothalamic-pituitary-interrenal (HPI) activity in teleost fish, cleaner fish social stress, cooperation, and learning (Winberg et al., 1997; Paula et al., 2015; Soares et al., 2016; Winberg and Thörnqvist, 2016; Backström and Winberg, 2017). A third neurotransmitter, associated with acute stress in multiple species of fish, as reviewed by Gamperl et al. (1994) is norepinephrine. The role of norepinephrine in association with cleaner fish behavior and physiological change to client interaction is nonetheless undetermined.

Individual variation in wild parental brood fish during rearing, and a breeding program in early development, both emphasize the importance of investigating lumpfish responses to Atlantic salmon. While acute responses in lumpfish exposed to Atlantic salmon or salmon sensory cues have been investigated to evaluate behavioral and physiological plasticity (Staven et al., 2019, 2021), the following study aimed to investigate how lumpfish responded to the presence of Atlantic salmon or salmon sensory cues over a period of 6 weeks. We measured physiological parameters relevant to stress and habituation including (1) growth, (2) health score assessment, (3) body color, (4) neurotransmitters, and (5) HPI axis responses.

## Materials and Methods

### Ethics Statement

Use of research animals was accepted for experimental purposes by the Norwegian Food Safety Authority (FDU #17231). Animals were carefully handled based on the Norwegian law on Regulation of Animal Experimentation (FOR-1996-01-15-23). Personnel involved were certified with FELASA-C, developed by the Federation of European Laboratory Animal Science Association. The experiment was conducted based on the three R’s and the ARRIVE guidelines (Kilkenny et al., 2010).

### Research Animals and Tagging

#### Lumpfish

Lumpfish used in the study originated from wild mature lumpfish captured in Troms and Finnmark county, Norway. Milt and roe were collected, fertilized, hatched, and reared from February 2018 at Mørkvedbukta AS. Lumpfish were fed with pellets based on standard recommendations given by a commercial feed producer (Skretting, Stavanger, Norway). All lumpfish were vaccinated with AMarine micro 4-2^®^ (Pharmaq, Overhalla, Norway) and given 300 day-degrees immunization before they were transferred to the Mørkvedbukta research station. Lumpfish used were juvenile individuals with low gonadal development, indicating no maturation. Lumpfish were tagged 1 week prior to experiment start with Floy tag t-bars (Floy Tag and Mfg Inc., Seattle, WA, United States). Tags were attached in the dorsal crest using a t-bar pistol after fish had been anesthetized with 100 mg l^–1^ benzocaine (ACD Pharmaceuticals, Leknes, Norway) based on recommendations in Skår et al. (2017).

#### Atlantic Salmon

Atlantic salmon used in the study were produced from an 11th generation Aquagen strain, hatched at Sundsfjord Smolt AS in Nordland, Norway. Smolts were transported to Mørkvedbukta research station in Bodø during the spring of 2017. At the station, feeding was conducted in same manners as later during the experiment. This included the use of automatic feeders (Arvo-Tec Oy, Huutokoski, Finland) containing Gemma diamond 150 (Skretting, Stavanger, Norway) split across three dosages adding up to a daily amount of 2% biomass. A total of eight Atlantic salmon (246.7 g, S.D ± 25.50) were used in the experiment and had no previous experience with lumpfish.

### Experimental Preparation

On the first day of the experiment (October 30, 2018), 160 lumpfish (39.7 g, S.D ± 8.99) were morphologically measured (see section “Growth”), photographed (see section “Skin Coloration”), and health assessed (see section “Health Assessment”). Next, lumpfish were randomly distributed in eight gray tank units (1 × 1 × 1 m, 480 L) with lids to avoid visual disturbance. With three different treatments in addition to a control group, the distribution of lumpfish were 20 individuals per tank with two tanks per treatment (*n* = 40). During the 6-week experimental period, the water flow in each tank was 500 L h^–1^ while mean ± SD oxygen saturation and water temperature from all eight tanks was 91.6 ± 1.84% and 7.5 ± 0.07°C, respectively. The photoperiod during the experiment was 24:0. Fish were fed 2% of their biomass separated into three meals served 08:00, 11:00, and 14:00 with 30 doses of pellets for each meal (Arvo-Tec Oy, Finland). Spilled feed and feces were removed once a week, but not within 4 days prior to sampling dates to avoid disturbance. Every second week, each individual lumpfish was morphologically measured and assessed for health scores. To avoid stress during handling, fish were quickly hand netted from the experimental tank to a 30 L tank with 5 mg L^–1^ of cortisol blocking metomidate (Aquacalm™, Western Chemical Inc, Ferndale, United States). This concentration causes rapid anesthesia and stops further increase of plasma cortisol (Iversen et al., 2003; Iversen and Eliassen, 2014). Fish were moved back to their respective tanks after the measurements were done.

### Treatments

The different treatments in the duplicate tanks were named “Live,” “Model,” and “Olfaction.” The duration of the treatments was 6 weeks. Two remaining tanks (“Control”) contained lumpfish exposed to no specific treatment. The Live treatment included cohabitation with two Atlantic salmon per tank. Model treatments involved two silicone salmonid models (*3D Line Thru 15 cm*, Savage Gear™, United States). Models were the same size as the initial size of Atlantic salmon used in the Live treatment. Each salmon model was attached to a nylon string and a swivel above the tank, which caused the models to move with the water current in the surface center area of the tank during the entire experiment. In the Olfaction treatment, the water input was connected to an adjacent tank containing four Atlantic salmon. Without disturbing flow or environmental parameters, the duplicate tanks were provided with a stable influx of water representing the presence of Atlantic salmon without any visual cues.

### Data Analysis

#### Growth

Weight (*W*) was measured with 0.1 g accuracy, while length (*L*) was measured to the nearest 0.1 cm, one fish at the time. Specific growth rates (SGR % day^–1^) were calculated using the formulae developed by Houde and Schekter (1981):


$$SGR=\left(e^{g}-1\right)*100$$

where *g* = [ln(*W*_2_) − ln(*W*_1_)/(t_2_ − t_1_)]. *W*_2_ refers to weight on the last day of measurement for the respective period, while *W*1 refers to weight on the first day. t_2_ and t_1_ refer to the time period described as number of days.

Formula for condition factor (*K*) was:


$$K=100*W/L^{3}$$

It was decided to use SGR and K-factor in the present study to compare findings with previous studies on lumpfish involving growth (Nytrø et al., 2014; Jørgensen et al., 2017; Imsland et al., 2018b, 2019).

#### Skin Coloration

Skin color analysis of lumpfish epidermal skin was assessed using methods described in Staven et al. (2021). Each lumpfish was photographed, and later analyzed for pixel counts within a defined area of the lateral side of the fish. Mean values of each of the three primary colors red (R), green (G), and blue (B) were measured using the histogram function in ImageJ, version 1.53e (NIH, Bethesda, MD, available at https://imagej.nih.gov/ij/). Eventually, the mean value for each of the three colors were added together, referred to as mean R + G + B.

#### Health Assessment

External health was scored based on health assessment criteria specifically developed for lumpfish (Imsland et al., 2020). This involved scoring (from 0 to 4) body conditions, tail fins, other fins, deformities, cataract, eye ulceration and the condition factor (*K*). For each parameter, a lower score indicated less deviations from normal health conditions. Individual scores were summated and the mean score per treatment calculated. Mean welfare score from 0 to 11 suggested “no to minimal deterioration,” while mean welfare score from 11 to 16 suggested “higher incidence of compromised health.” A score above 16 indicated “evidence of further extensive health deterioration.” For a detailed scoring description, see Imsland et al. (2020).

#### Blood and Brain Sampling for Plasma Cortisol and Neurotransmitters

Blood and brains were sampled after 6 weeks of experiment. To avoid activation of the HPI-axis during the final handling, water supply was stopped and 5 mg L^–1^ metomidate (Aquacalm™, Western Chemical Inc., Canada) quickly added to one tank at the time. Amnesia (hypnosis) occurred within 2 min in lumpfish. Next, fish were removed from the tank with a hand net before blood was sampled from the caudal vessel using 0.33 × 12.7 mm syringes (BD Micro-fine^®^). Anticoagulating heparin (Leo Pharma, Malmø, Sweden) was added to the syringes by pulling the plunger out and back again into its initial position, thus leaving a small amount of heparin in the dead space of the syringe (approximately 0.08 mL). Samples were centrifuged at 5000 RPM (2000 × *g*) for 10 min in a Mini Star centrifuge (VWR™, United Kingdom). After centrifugation, the plasma was separated with a pipette, transferred to a 1.8 mL Nunc Cryo Tube^®^ and stored at −40°C. Plasma cortisol levels were later analyzed using radioimmunoassay, based on methods described in Iversen et al. (1998). Next, fish were euthanized by spinal transection and gills were cut. Left hemisphere brains were removed, separated along the sagittal plane, and stored on dry ice, while right hemisphere brains were designated for other experiments and not included in the study. Concentrations of norepinephrine (NE), dopamine (DA), 3,4-dihydroxyphenylacetic acid (DOPAC), serotonin (5-hydroxytryptamine, 5-HT), and 5-hydroxyindoleacetic acid (5-HIAA) in left hemisphere brain tissue were later quantified by means of high performance liquid chromatography (HPLC) with electrochemical detection as previously described (Alfonso et al., 2019).

#### Stimulation and Suppression Test of Hypothalamic-Pituitary-Interrenal Axis

To investigate treatment impact on the HPI-axis feedback system, a stimulation and suppression test using adrenocorticotropic hormone (ACTH) and dexamethasone (DEX), respectively, was conducted in accordance with the previous study by Pottinger and Carrick (2001), with some minor modifications as described in Iversen and Eliassen (2014).

## Statistics

All statistical tests were performed using the R software™ R.3.2.2 (R Development Core Team, 2013) at a 95% level of significance. Data were tested for normality using the Shapiro–Wilk test (Shapiro and Wilk, 1965) while variance was tested using Levene’s *F*-test (Gastwirth et al., 2009). Treatments and control were analyzed with a one-way ANOVA test. When assumptions of normality or homoscedasticity were not met, a non-parametric Kruskal–Wallis test were used instead. If significant differences occurred, a *post hoc* test was used to compare treatments and control with one another. A Tukey test was used after one-way ANOVA, while a Dunn test was used after a Kruskal–Wallis test. Results were presented using mean values ± standard deviation (mean ± S.D). Figures were graphically depicted as bar graphs with means and 95% confidence intervals, or as boxplots with a median central line and whiskers covering 95% of values, while separate dots indicated outliers. Letters were used to illustrate significant differences from *post hoc* tests, while no letters indicated no significant difference from the ANOVA or Kruskal–Wallis tests. From 20 lumpfish in each experimental tank, 12 were used for real time measurements of plasma cortisol, including six lumpfish also used for measurements of brain neurotransmitters. The remaining eight in each tank were used for stimulation and suppression test of HPI axis.

## Results

### Morphometric Parameters

#### Growth and Condition of Lumpfish

**FIGURE 1:**
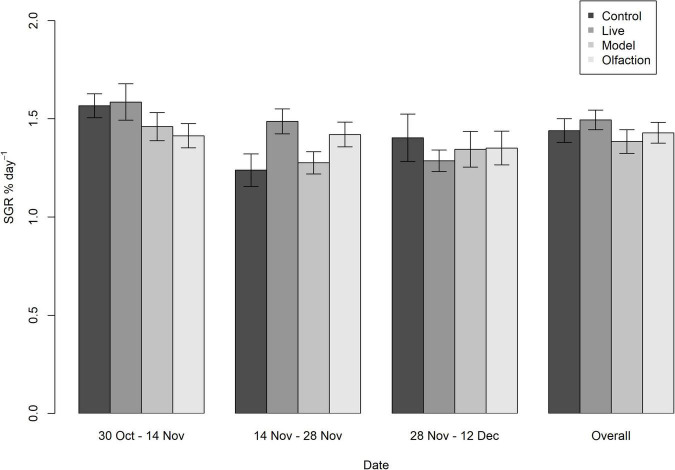
Specific growth rate (SGR % day^–1^) in lumpfish (*n* = 40 per treatment) during 6 weeks of interaction with Live, Model or Olfaction treatments or Control. Mean measurements with 95% confidence interval error bars revealed no difference between treatments measured every second week, including an overall measurement comparing initial weight with endpoint weight.

Initial measurements of mean weight did not differ between treatments and control [*F*(3,157) = 0.950, *p* = 0.44]. During the experiment, specific growth rates did not differ between treatments after 2 weeks [*K*(158) = 159, *p* = 0.467], 4 weeks [*K*(158) = 159, *p* = 0.485], 6 weeks [*K*(158) = 159, *p* = 0.485], or overall [*K*(160) = 160, *p* = 0.485; Figure 1]. Also, the condition factors (*K*) measured after experimental termination in Control (3.93 ± 0.44), Live (3.93 ± 0.34), Models (4.01 ± 0.44), and Olfaction (3.91 ± 0.48) did not significantly differ [*F*(3,157) = 0.388, *p* = 0.76].

#### Mortality and Health Score Assessments

No mortality was observed during the experiment. Health score assessments done after the experiment was terminated, revealed no visible damage (score = 0) for mouth, skin, gills, fins, or eyes in all fish, with exception of a single fish in the Live treatment, that had a skin score of 1. Skin penetration in the dorsal crest from Floy tag t-bars did not cause wounds. Mean condition factor (*K*) for each treatment, including Control, were within the score range of 3.5–4.5, which was defined as in “moderate condition,” giving a health score of “1.”

#### Skin Coloration

**FIGURE 2:**
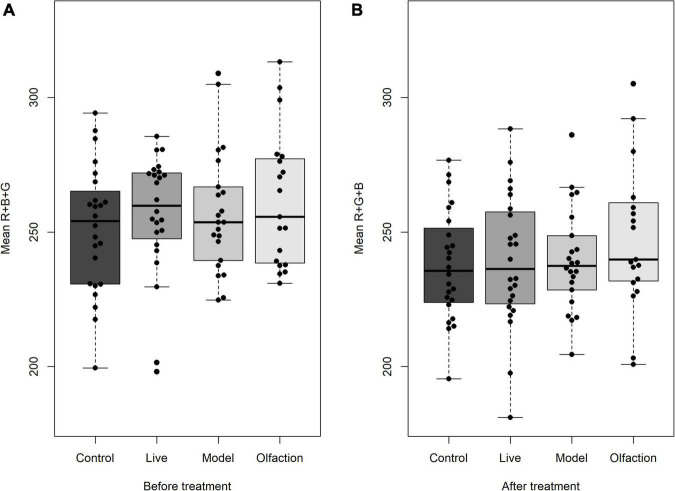
Lumpfish skin color (*n* = 24) measured as mean Red + Green + Blue pixels **(A)** before and **(B)** after treatment exposure. Mean R + G + B did not deviate between treatments before or between treatments after the experiment. Lumpfish were reared in experimental tanks with Live Atlantic salmon or different salmon sensory cues (Olfaction and Model). Boxplots describe median values with quantiles and outliers.

Skin coloration measured on the first day of experiment did not differ when comparing mean R + G + B in treatments and control [*F*(3,85) = 1.35, *p* = 0.261; Figure 2A]. Similarly, the 6 weeks of cohabitation did not induce any change in mean R + G + B measurements between treatments and control [*F*(3,85) = 0.727, *p* = 0.539; Figure 2B].

### Neurotransmitters

#### Dopamine

**FIGURE 3:**
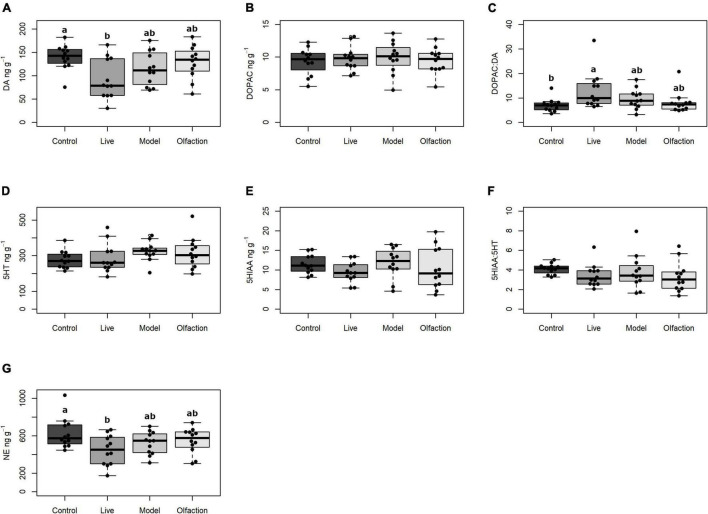
The levels of neurotransmitters (ng g^–1^) in ft hemisphere of brains from lumpfish (*n* = 12) exposed to Live, Model, or Olfaction treatments in addition to a Control. Neurotransmitters include **(A)** DA (dopamine), **(B)** DOPAC (3,4-dihydroxyphenylacetic acid), **(C)** the DOPAC:DA turnover ratio, **(D)** 5HT (serotonin), **(E)** 5HIAA (5-hydroxyindoleacetic acid), **(F)** the 5HIAA:5HT turnover ratio, and **(G)** NE (Norepinephrine). Treatments that share a letter were not significantly different (*p* < 0.05).

The levels of dopamine (DA) in treatments and control were significantly different [*F*(3,44) = 3.97, *p* = 0.013] and a *post hoc* comparison revealed significantly lower concentrations (*p* = 0.010) in Live (92.7 ± 42.60 ng/g) compared to Control (140.2 ± 27.26 ng/g). The dopaminergic activity index (the DOPAC:DA ratio) was significantly different between treatments and control [*H*(3) = 9.67, *p* = 0.021], and a *post hoc* comparison revealed significant increase (*p* = 0.002) of the DOPAC:DA ratio in the Live treatment (DOPAC:DA = 13.0% ± 7.58) compared to the Control (DOPAC:DA = 7.0% ± 2.70). Levels of the metabolite 3,4-dihydroxyphenylacetid acid (DOPAC) did not differ between treatments and control [*F*(3,44) = 0.21, *p* = 0.889; Figures 3A–C].

#### Serotonin

No difference between treatments and control were observed for serotonin (5-HT) [*F*(3,44) = 1.26, *p* = 0.3], the serotonin metabolite (5HIAA) [*F*(3,44) = 1.03, *p* = 0.387] or the serotonergic activity index (5HIAA:5-HT) [*F*(3,44) = 0.94, *p* = 0.425] (Figures 3D–F).

#### Norepinephrine

A significant difference in levels of norepinephrine (NA) was observed between treatments and control [*F*(3,44) = 3.08, *p* = 0.036; Figure 3G], and a *post hoc* test showed that the difference (*p* = 0.021) occurred between Live (455.8 ± 159.49) and Control (624.4 ± 170.64).

### Hypothalamic-Pituitary-Interrenal Axis Responses

#### Basal Levels

**FIGURE 4:**
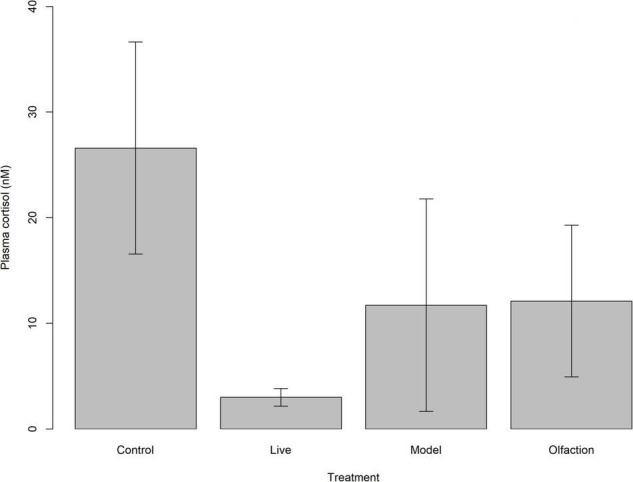
Mean basal plasma cortisol (nM) levels with 95% confidence intervals in lumpfish (*n* = 24) after 6 weeks of treatment exposure. Treatments involved interaction with two Atlantic salmon (Live), two salmon models (Model) or salmon smell from a tank containing four Atlantic salmon (Olfaction).

Basal levels of plasma cortisol concentrations were similar between lumpfish (*n* = 24) in treatments and control [*H*(3) = 6.23, *p* = 0.1]. Mean ± SD plasma cortisol for the treatments were lowest in Live (3.0 ± 3.92 nmol L^–1^) and highest in Control (26.6 ± 48.11 nmol L^–1^), while Model and Olfaction were 11.7 ± 49.24 nmol L^–1^ and 12.1 ± 35.15 nmol L^–1^, respectively (Figure 4).

#### Stimulation and Suppression Test of Hypothalamic-Pituitary-Interrenal Axis

**FIGURE 5:**
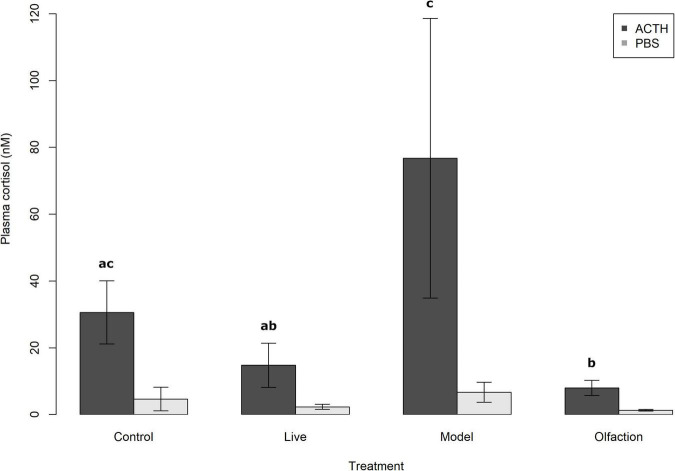
Mean plasma cortisol (nM) levels with 95% confidence intervals in lumpfish (*n* = 16) 2 h post-intraperitoneal injection with either 0.5 mL kg^–1^ phosphate-buffered saline (PBS) or 45 μg mL^–1^ adrenocorticotropic hormone (ACTH). 24 h prior to sampling, lumpfish were injected with 1 mg kg^–1^ dexamethasone in ethanol: PBS; 1:3; 1 μg L^–1^ (DEX) and relocated to dark tanks with the same environmental conditions as during the experiment. Treatments involved interaction with Live Atlantic salmon, salmon Models or salmon smell from a tank containing four Atlantic salmon. Treatments that share a letter were not significantly different (*p* < 0.05).

The remaining lumpfish (*n* = 16) per treatment that were used to evaluate the stimulation and suppression of the HPA axis showed no significant difference in plasma cortisol levels after phosphate-saline injection (PBS) [*H*(3) = 7.11, *p* = 0.07], while injection with adrenocorticotrophic hormone (ACTH) revealed different responses between treatments and control [*H*(3) = 11.51, *p* = 0.009; Figure 5]. A *post hoc* test found significant differences between Model (76.7 ± 118.40 nmol L^–1^) and Live (14.7 ± 18.60 nmol L^–1^) (*p* = 0.006), Model and Olfaction (8.0 ± 6.43 nmol L^–1^) (*p* = 0.001), and Olfaction and Control (30.6 ± 26.73 nmol L^–1^) (*p* = 0.017).

## Discussion

The study evaluated physiological effects in lumpfish exposed to Atlantic salmon or to different salmon sensory cues over a duration of 6 weeks. Live, Model, and Olfaction treatments induced no change in growth, body coloration, health assessments, or basal levels of plasma cortisol. Brain analysis revealed significant changes in concentrations of neurotransmitters observed in lumpfish cohabiting with Live Atlantic salmon. Both dopamine and norepinephrine concentrations were significantly lower in the Live treatment compared to Control. The stimulation and suppression test of the HPI axis found oversensitivity from the ACTH injection in one lumpfish from the Model treatment, while the Live and Olfaction treatments did not show any indications of oversensitivity. The present findings coincide with previously observed physiological acute impacts on lumpfish exposed to the same, or similar treatment protocols, and adds novel perspectives on how the species coexist over longer time periods without impeding the resting state or the welfare of the species (Staven et al., 2019, 2021). Below, we elaborate each specific investigated parameter.

### Growth

In aquaculture, specific growth rate (SGR) and the condition factor (*K*) can be used as indicators on the wellbeing of farmed fish (North et al., 2006; Calabrese et al., 2017). Deviations from a normal resting state can alter the metabolic scope and energy expenditure in fish due to the relocation of energy surpluses (e.g., gluconeogenic effect of corticosteroids) (Barton and Iwama, 1991). In lumpfish, SGR has been previously studied in relationship with temperature and fish size (Nytrø et al., 2014), families (Imsland et al., 2021), diet types (Imsland et al., 2019), water exchange, and oxygen saturation (Jørgensen et al., 2017). In the present study we investigated if predator cues could impact lumpfish to an extent where growth was affected. After 6 weeks of interaction, lumpfish showed no change in SGR or condition factors (*K*) from salmon sensory cues or cohabitation with Atlantic salmon in comparison with the Control. In other prey fish, or fish relatively small, the presence of predatory cues caused reduced growth performance (Strand et al., 2007; Sunardi et al., 2007) or impairments on the capacity of prey fish to obtain and preserve energy resources (Lima and Dill, 1990). When lumpfish are utilized in aquaculture, the transition from rearing conditions with conspecifics only to interaction with large Atlantic salmon induces elevated levels of plasma cortisol, increased swimming activity, and avoidance behavior (Staven et al., 2019). A follow up study revealed that swimming activity rapidly increased upon exposure to salmon cues before dropping again within the first hour of interaction (Staven et al., 2021). In accordance with both Staven et al. (2019) and Staven et al. (2021), salmon cues can initiate moderate acute stress responses, while these responses diminished during a prolonged interaction and did not affect growth. Overall SGR in treatments were approximately 0.6 points lower in comparison with Nytrø et al. (2014). Both studies had similar light regimes, temperatures, and oxygen saturations, yet Nytrø et al. (2014) conducted an optimal feeding trial with more lumpfish per tank and lower initial individual variation in weight. This shows that SGR is a relatively stable measurement of expected growth in a tank experiment and thus a good welfare indicator based on the given environment. The overall importance of our findings on SGR and condition factor was the limited effect from Atlantic salmon or salmon cues alone.

### Skin Coloration

Lumpfish express variation in skin coloration both early in ontogeny and after maturation (Davenport and Thorsteinsson, 1989; Davenport and Bradshaw, 1995). While skin coloration and pigmentation have previously been observed to alter based on background color and hue (Davenport and Bradshaw, 1995), acute changes within an hour of interaction were previously detected in both parameters when juvenile lumpfish were exposed to Atlantic salmon olfaction (Staven et al., 2021). In the current study, lumpfish color (measured as mean R + G + B) did not differ between any of the three treatments compared to Control. Acute stress has been associated with mediation and aggregation of melanosomes inside melanophore cells in fish, regulating both color and darkening (Fujii, 2000; Aspengren et al., 2003; Nilsson Sköld et al., 2013). Given that mechanisms of color change require energy, the physiological process of habituation to salmon cues would cause an overall reduction in unnecessary energy expenditure in lumpfish. Overall, the long-term interaction with salmon cues revealed a novel understanding of skin coloration change from social interaction and suggests that lumpfish induce little visual alterations when the resting state is not disrupted, while acute stress responses can increase both darkening and the vividness of the skin (Staven et al., 2021).

### Health Assessment and Mortality

Health scores were assessed before, during, and after the experiment based on methods described in Imsland et al. (2020). The experiment did not cause any deterioration in health score from interaction with the different salmon cues in comparison to the health scores observed in Control. This is the first experiment to measure the effect on health for lumpfish in interaction with Atlantic salmon or exposure to salmon sensory cues in a confined tank experiment. Previous studies on health scores in lumpfish were conducted in commercial scale sea pens together with Atlantic salmon and were not designed to measure solely the effect from salmon interaction or salmon cues (Imsland et al., 2020; Gutierrez Rabadan et al., 2021). In general, the little negative impacts on health scores were in accordance with the physiological measurements. With no clinical or physiological indications of negative impacts from treatments, zero mortalities in lumpfish were as expected.

### Neurotransmitters

Monoaminergic neuromodulators have important roles in the regulation of different physiological and behavioral processes, both in vertebrates and invertebrates. This includes learning and memory, wakefulness and arousal, stress responses, social interactions, and aggression, among others (Swallow et al., 2016; Ranjbar-Slamloo and Fazlali, 2020). Recent studies have shown that brain monoamines might be important in regulating interspecific patterns of behavior such as those between cleaner fish and their clients (Soares, 2017; de Abreu et al., 2018). In our study, the 6-week cohabitation with Live salmon induced a decrease in the levels of catecholamines, dopamine, and norepinephrine in the brain of the lumpfish. Both compounds share a biosynthetic route from the amino acid tyrosine and seem to functionally overlap to a certain extent in vertebrates (Ranjbar-Slamloo and Fazlali, 2020). The dopaminergic system in fish is known to be involved in processes such as locomotor activity, learning, motivation, reward, reproduction, stress, and social behavior including aggression, dominance, and interspecific interactions, as observed between cleaner and client fish (Summers and Winberg, 2006; Gesto et al., 2013; Soares, 2017; Ganesh, 2021). Among those functions, both stress-related factors and the social interaction with the introduced salmon might have been involved in the observed changes in the lumpfish brain. Furthermore, based on the results of this and a previous study (Staven et al., 2021), the social factor was likely the most important. Social factors were considered the most relevant because stress related alterations in the brain monoaminergic systems are usually more prominent in the serotonergic than in the dopaminergic system, both in situations of acute and prolonged stress (Øverli et al., 2001; Gesto et al., 2013; Conde-Sieira et al., 2018; López-Patiño et al., 2021), and no alterations were found in the serotonergic system in this study. Furthermore, our previous study (Staven et al., 2021) showed that both Live and Olfaction treatment were equally able to acutely modify the swimming activity of the lumpfish (in a kind of “startling” response), while only the Live treatment significantly affected the lumpfish brain in the current study. Even if the observed brain alterations could be the result of social factors related to the presence of salmon, the precise physiological significance of the reduced levels of both dopamine and norepinephrine in the lumpfish exposed to live salmon are difficult to interpret. Reduced levels of the parent catecholamines could be the result of a reduced synthesis rate, a sustained higher release and subsequent oxidation, or both. In the Live group, despite the reduction in dopamine, the DOPAC levels remained unaltered, thus resulting in a higher DOPAC/DA turnover ratio. The higher turnover ratio suggests that DAergic neurons had a higher firing rate in the Live group. Acute changes in the dopamine system have been observed before in specific brain areas upon acute exposure to stress or social interaction (Gesto et al., 2013; Teles et al., 2013; de Abreu et al., 2018). However, the comparison of the current data with those studies is complex since we did not perform a brain region-specific analysis. Furthermore, prolonged exposure to the same events might result in different alterations, since the brain will have time to finely modulate the release of neurotransmitters to adjust to the new situation by using different feedback mechanisms (Best et al., 2009, 2010). In addition, prolonged exposure periods would include potential effects of other brain processes related to memory, learning, and motivation/reward mechanisms to adjust fish behavior to the new situation. In this regard, it can be even questioned whether the turnover ratios (metabolite/amine) are still good estimators of neuron firing rates, since other factors could be having a relevant effect on the levels of the metabolite; for example, alterations in the monoamine oxidase activity cannot be ruled out (Higuchi et al., 2017, 2019). Interestingly, a long-term increase in overall brain catecholaminergic activity was observed in gilthead seabream after early life exposure to stress for 2 weeks (Vindas et al., 2018). In that study, however, the DAergic ratio was increased in the brain because of increased levels of the metabolite DOPAC, and not because of a decrease in dopamine amounts, so the mechanism behind the alterations is likely different. Together with the lack of differences in terms of growth performance and health scores, brain data suggest that lumpfish were able to cope with the treatments, and only the Live group required some adjustment in the brain pathways involved in behavioral regulation. The fact that dopamine adjustments were observed only in Live and not in the Model or Olfaction treatments, demonstrate that single cues were not having a relevant effect on the lumpfish brain in the long term, also supporting the view that dopamine alterations were likely the result of interspecific social contact.

### Effects on the Hypothalamic-Pituitary-Interrenal Axis

In mammals, several changes in the HPI-axis have been documented during a chronic stress state. This includes weight loss, proliferation of the corticotrope cells in the anterior pituitary, inhibition of ACTH synthesis by cortisol, reduction of the feedback effect of glucocorticoid receptor agonists on ACTH release, increase of the size of the adrenal glands and of the response of the adrenals to ACTH (Mormède et al., 2007). This resetting of the HPI axis at a new level of activity (set points), that Selye (1975) described as the stage of resistance, is also known as allostasis (McEwen, 1998; McEwen and Wingfield, 2003; Goymann and Wingfield, 2004; Wingfield, 2005). Different approaches can be used to detect these changes as described above, including stimulation tests (activating corticotropic releasing hormone, vasopressin, ACTH, and insulin-induced hypoglycemia) that measure the relative sensitivity of the pituitary and the interrenal cells, and the use of an inhibition test utilizing DEX to demonstrate the reduced efficiency of the negative feedback by corticosteroids (Mormède et al., 2007). In this experiment, the treatment exposure induced variations in mean concentrations of plasma cortisol after administration of a weight-adjusted dose of ACTH. At first observation, lumpfish exposed to Models revealed oversensitivity to administration of a weight-adjusted dose of ACTH compared to the other treatments. Data unveiled a single outlier (strong stress response of 366.85 nM), which increased mean plasma cortisol level of the Model treatment from 18.34 to 32.86 nM. While removal of the outlier would have impacted the statistical outcome, resulting in no significant difference between Model, Olfaction and Live treatments, individual variation in stress responses in fish should be accounted for. This is due to the reality of different behavioral strategies observed between individuals within the same species referred to as bold or shy, proactive, or reactive, or as differences in personalities (Schjolden and Winberg, 2007; Toms et al., 2010; Yuan et al., 2018). In lumpfish, differences in personality have recently been observed when individuals were exposed to novel objects and later introduced to Atlantic salmon (Whittaker et al., 2021). Variation in stress responses in lumpfish during novel heterospecific interactions could be associated with why only certain lumpfish graze sea lice when deployed in net pens with Atlantic salmon, especially in the context of neurotransmitters associated with cooperative establishment and social behavior. Overall, few studies on ACTH sensitivity have been done on fish. However, Pottinger and Carrick (2001) showed that two strains of rainbow trout selected for high (HR) and low (LR) responsiveness to a standard crowding test had different responsiveness to a weight-adjusted dose of ACTH. The LR strain had significant lower production of plasma cortisol compared to the HR strain. In domesticated mammals, an injection of ACTH has shown an increased cortisol response in animals reared in poor conditions or subjected to repeated stressors. Similarly, Iversen and Eliassen (2014) showed that salmon that were stressed daily over a 4-week period became oversensitive to ACTH, regardless of whether the fish were vaccinated before or after applied stressor. One can only speculate how the Model treatment triggered the HPI axis in a single individual. However, the models used were novel, and the introduction of novel objects is a common method used to distinguish different individual responses in fish (Castanheira et al., 2013; White et al., 2013; Whittaker et al., 2021). Exposure to Models were not enough to elicit at full blown allostasis type 2 response, referred to as chronical stress, recognized by an oversensitive ACTH axis linked with non-functioning negative feedback (DEX) as described by Iversen and Eliassen (2014). Plasma cortisol measured in lumpfish sampled from the experimental tanks after 6 weeks of treatment exposure revealed no significant difference between the different treatments and the control. Still, a tendency of reduced plasma cortisol levels in the Live treatment was observed, similar what was seen as reduced concentrations of norepinephrine and dopamine after coexisting with Atlantic salmon. The combination of the stimulation and suppression test together with overall low basal levels of plasma cortisol suggested that treatments and interaction with Atlantic salmon induced a plastic change in lumpfish where the threshold of activation of the HPI-axis was downregulated.

## Conclusion

We investigated how lumpfish responded to the presence of carnivorous Atlantic salmon or salmon sensory cues through analysis of multiple physiological parameters relevant to welfare, social interaction, and stress during a 6-week tank experiment. Results showed that growth, health scores, and skin coloration remained unaltered. The stimulation and suppression test of HPI-axis and the overall low plasma cortisol levels suggested no indication of allostasis type 2 chronic stress or acute stress, respectively. Interaction with live salmon induced alterations in the brain of the lumpfish, which revealed reduced levels of brain catecholamines, namely norepinephrine and dopamine. The cause and function of these alterations are not completely understood but could be part of a necessary process to adjust lumpfish behavior to cohabiting with free-roaming salmon. Lumpfish behavior was not assessed during the experiment, and a future focus on potential relationships between altered neurotransmission and behavioral adjustment would be necessary to test if that was the case. The novel findings on the plastic change in neuromodulators could be utilized in future work identifying targets for breeding selection, considering the importance of their role in social interaction in other species of cleaner fish. Overall, this study showed that lumpfish were not negatively impacted by cohabitation or exposure to salmon cues, which suggested that welfare disruption in commercial production is probably related to a combination of Atlantic salmon exposure with other stressors.

## Data Availability Statement

The raw data supporting the conclusions of this article will be made available by the authors, without undue reservation.

## Ethics Statement

The animal study was reviewed and approved by Norwegian Food Safety Authority (FDU#17231).

## Author Contributions

FS, TK, and JN contributed to the design of the study. FS conducted the study with help from DP and PA and made the first manuscript draft. MG and MI were in charge of brain analysis and cortisol measurements, and wrote sections of the manuscript. All authors contributed to manuscript revision, read, and approved the submitted version.

## Conflict of Interest

FS and PA were employed by Aqua Kompetanse AS. The remaining authors declare that the research was conducted in the absence of any commercial or financial relationships that could be construed as a potential conflict of interest.

## Publisher’s Note

All claims expressed in this article are solely those of the authors and do not necessarily represent those of their affiliated organizations, or those of the publisher, the editors and the reviewers. Any product that may be evaluated in this article, or claim that may be made by its manufacturer, is not guaranteed or endorsed by the publisher.

## References

[B1] Alfonso S. Sadoul B. Gesto M. Joassard L. Chatain B. Geffroy B. (2019). Coping styles in European sea bass: the link between boldness, stress response and neurogenesis. *Physiol. Behav.* 207 76–85. 10.1016/j.physbeh.2019.04.020 31047951

[B2] Aspengren S. Sköld H. N. Quiroga G. Mårtensson L. Wallin M. (2003). Noradrenaline- and melatonin-mediated regulation of pigment aggregation in fish melanophores. *Pigm. Cell Res.* 16 59–64. 10.1034/j.1600-0749.2003.00003.x 12519126

[B3] Backström T. Winberg S. (2017). Serotonin coordinates responses to social stress - what we can learn from fish. *Front. Neurosci.* 11:595. 10.3389/fnins.2017.00595 29163002 PMC5669303

[B4] Barton B. A. Iwama G. K. (1991). Physiological changes in fish from stress in aquaculture with emphasis on the response and effects of corticosteroids. *Annu. Rev. Fish Dis.* 1 3–26. 10.1016/0959-8030(91)90019-G

[B5] Berejikian B. A. Tezak E. P. LaRae A. L. (2003). Innate and enhanced predator recognition in hatchery-reared chinook salmon. *Environ. Biol. Fishes.* 67 241–251. 10.1023/A:1025887015436

[B6] Best J. Nijhout H. F. Reed M. (2010). Serotonin synthesis, release and reuptake in terminals: a mathematical model. *Theor. Biol. Med. Model.* 7:34. 10.1186/1742-4682-7-34 20723248 PMC2942809

[B7] Best J. A. Nijhout H. F. Reed M. C. (2009). Homeostatic mechanisms in dopamine synthesis and release: a mathematical model. *Theor. Biol. Med. Model.* 6:21. 10.1186/1742-4682-6-21 19740446 PMC2755466

[B8] Bjordal Å (1990). Sea lice infestation on farmed salmon: possible use of cleaner-fish as an alternative method for de-lousing. *Can. Tech. Rep. Fish. Aquat. Sci.* 1761 85–89.

[B9] Blanco Gonzalez E. de Boer F. (2017). The development of the norwegian wrasse fishery and the use of wrasses as cleaner fish in the salmon aquaculture industry. *Fish. Sci.* 83 661–670. 10.1007/s12562-017-1110-4

[B10] Brown G. E. (2003). Learning about danger: chemical alarm cues and local risk assessment in prey fishes. *Fish Fish.* 4 227–234. 10.1046/j.1467-2979.2003.00132.x

[B11] Brown G. E. Godin J. G. J. (1999). Who dares, learns: chemical inspection behaviour and acquired predator recognition in a characin fish. *Anim. Behav.* 57 475–481. 10.1006/anbe.1998.1017 10049488

[B12] Brown G. E. Rive A. C. Ferrari M. C. O. Chivers D. P. (2006). The dynamic nature of antipredator behavior: prey fish integrate threat-sensitive antipredator responses within background levels of predation risk. *Behav. Ecol. Sociobiol.* 61 9–16. 10.1007/s00265-006-0232-y

[B13] Bshary R. (2001). “The cleaner fish market,” in *Economics in Nature*, eds Noë R. Van Hooff J. A. R. Hammerstein P. (Cambridge: University Press), 146–172. 10.1086/707519

[B14] Bshary R. Würth M. (2001). Cleaner fish (*Labroides dimidiatus*) manipulate client reef fish by providing tactile stimulation. *Proc. R. Soc. Lond.* 268 1495–1501. 10.1098/rspb.2001.1495 11454294 PMC1088769

[B15] Calabrese S. Nilsen T. O. Kolarevic J. Ebbesson L. O. E. Pedrosa C. Fivelstad S. (2017). Stocking density limits for post-smolt Atlantic salmon (*Salmo salar* L.) with emphasis on production performance and welfare. *Aquaculture* 468 363–370. 10.1016/j.aquaculture.2016.10.041

[B16] Castanheira M. F. Herrera M. Costas B. Conceição L. E. C. Martins C. I. M. (2013). Can we predict personality in fish? Searching for consistency over time and across contexts. *PLoS One* 8:e62037. 10.1371/journal.pone.0062037 23614007 PMC3628343

[B17] Chivers D. P. Smith R. J. F. (1998). Chemical alarm signalling in aquatic predator-prey systems: a review and prospectus. *Écoscience* 5 338–352. 10.1080/11956860.1998.11682471

[B18] Clinchy M. Sheriff M. J. Zanette L. Y. (2013). Predator-induced stress and the ecology of fear. *Funct. Ecol.* 27 56–65. 10.1111/1365-2435.12007

[B19] Conde-Sieira M. Valente L. M. P. Hernández-Pérez J. Soengas J. L. Míguez J. M. Gesto M. (2018). Short-term exposure to repeated chasing stress does not induce habituation in *Senegalese sole*, *Solea senegalensis*. *Aquaculture* 487 32–40. 10.1016/j.aquaculture.2018.01.003

[B20] Costello M. J. (2009). The global economic cost of sea lice to the salmonid farming industry. *J. Fish. Dis.* 32 115–118. 10.1111/j.1365-2761.2008.01011.x 19245636

[B21] Davenport J. Bradshaw C. (1995). Observations on skin colour changes in juvenile lumpsuckers. *J. Fish. Biol.* 47 143–154. 10.1111/j.1095-8649.1995.tb01880.x

[B22] Davenport J. Thorsteinsson V. (1989). Observations on the colours of lumpsuckers, *Cyclopterus lumpus* L. *J. Fish. Biol.* 35 829–838. 10.1111/j.1095-8649.1989.tb03034.x

[B23] de Abreu M. S. Maximino C. Cardoso S. C. Marques C. I. Pimentel A. F. N. Mece E. (2020). Dopamine and serotonin mediate the impact of stress on cleaner fish cooperative behavior. *Horm. Behav.* 125:104813. 10.1016/j.yhbeh.2020.104813 32619442

[B24] de Abreu M. S. Messias J. P. M. Thörnqvist P.-O. Winberg S. Soares M. C. (2018). The variable monoaminergic outcomes of cleaner fish brains when facing different social and mutualistic contexts. *PeerJ* 6:e4830. 10.7717/peerj.4830 29844980 PMC5971103

[B25] Eaton R. C. Bombardieri R. A. Meyer D. L. (1977). The mauthner-initiated startle response in teleost fish. *J. Exp. Biol.* 66 65–81. 10.1242/jeb.66.1.65 870603

[B26] Eliasen K. Danielsen E. Johannesen Á Joensen L. L. Patursson E. J. (2018). The cleaning efficacy of lumpfish (*Cyclopterus lumpus* L.) in *Faroese salmon* (*Salmo salar* L.) farming pens in relation to lumpfish size and seasonality. *Aquaculture* 488 61–65. 10.1016/j.aquaculture.2018.01.026

[B27] Ferrari M. C. Wisenden B. D. W. Chivers D. P. C. (2010). Chemical ecology of predator - prey interactions in aquatic ecosystems: a review and prospectus. *Can. J. Zool.* 88 698–724. 10.1139/z10-029

[B28] Fujii R. (2000). The regulation of motile activity in fish chromatophores. *Pigment Cell Res.* 13 300–319. 10.1034/j.1600-0749.2000.130502.x 11041206

[B29] Gamperl A. K. Vijayan M. M. Boutilier R. G. (1994). Experimental control of stress hormone levels in fishes: techniques and applications. *Rev. Fish Biol. Fisher.* 4 215–255. 10.1007/BF00044129

[B30] Ganesh C. B. (2021). The stress – reproductive axis in fish: the involvement of functional neuroanatomical systems in the brain. *J. Chem. Neuroanat.* 112:101904. 10.1016/j.jchemneu.2020.101904 33278567

[B31] Gastwirth J. L. Gel Y. R. Miao W. (2009). The impact of Levene’s test of equality of variances on statistiacal theory and practice. *Stat. Sci.* 24 343–360. 10.1214/09-STS301

[B32] Gesto M. López-Patiño M. A. Hernández J. Soengas J. L. Míguez J. M. (2013). The response of brain serotonergic and dopaminergic systems to an acute stressor in rainbow trout: a time course study. *J. Exp. Biol.* 216 4435–4442. 10.1242/jeb.091751 24031060

[B33] Gingins S. Roche D. G. Bshary R. (2017). Mutualistic cleaner fish maintains high escape performance despite privileged relationship with predators. *Proc. R. Soc. B.* 284:2469. 10.1098/rspb.2016.2469 28424344 PMC5413917

[B34] Goymann W. Wingfield J. C. (2004). Allostatic load, social status and stress hormones: the costs of social status matter. *Anim. Behav.* 67 591–602. 10.1016/j.anbehav.2003.08.007

[B35] Grutter A. S. (1999). Cleaner fish really do clean. *Nature* 398 672–673. 10.1038/19443

[B36] Grutter A. S. (2004). Cleaner fish use tactile dancing behavior as a preconflict management strategy. *Curr. Biol.* 14 1080–1083. 10.1016/j.cub.2004.05.048 15203000

[B37] Gutierrez Rabadan C. Spreadbury C. Consuegra S. Garcia de Leaniz C. (2021). Development, validation and testing of an operational welfare score index for farmed lumpfish *Cyclopterus lumpus* L. *Aquaculture* 531:735777. 10.1016/j.aquaculture.2020.735777

[B38] Higuchi Y. Soga T. Parhar I. S. (2017). Regulatory pathways of monoamine oxidase a during social stress. *Front. Neurosci.* 11:604. 10.3389/fnins.2017.00604 29163009 PMC5671571

[B39] Higuchi Y. Soga T. Parhar I. S. (2019). Social defeat stress decreases mRNA for monoamine oxidase A and increases 5-HT turnover in the brain of male Nile tilapia (*Oreochromis niloticus*). *Front. Pharmacol.* 9:1549. 10.3389/fphar.2018.01549 30687104 PMC6333864

[B40] Houde E. D. Schekter R. D. (1981). Growth rates, rations and cohort consumption of marine fish larvae in relation to prey concentrations. rapp. P.-v. *Reun. Cons. Int. Explor. Mer* 178 441–453.

[B41] Imsland A. K. Reynolds P. Eliassen G. Hangstad T. A. Foss A. Vikingstad E. (2014a). The use of lumpfish (*Cyclopterus lumpus* L) to control sea lice (*Lepeophtheirus salmonis* Kroyer) infestations in intensively farmed Atlantic salmon (*Salmo salar* L). *Aquaculture* 424 18–23. 10.1016/j.aquaculture.2013.12.033

[B42] Imsland A. K. Reynolds P. Eliassen G. Hangstad T. A. Nytro A. V. Foss A. (2014b). Notes on the behaviour of lumpfish in sea pens with and without Atlantic salmon present. *J. Ethol.* 32 117–122. 10.1007/s10164-014-0397-1

[B43] Imsland A. K. Reynolds P. Eliassen G. Mortensen A. Hansen ØJ. Puvanendran V. (2016a). Is cleaning behaviour in lumpfish (*Cyclopterus lumpus*) parentally controlled? *Aquaculture* 459 156–165. 10.1016/j.aquaculture.2016.03.047

[B44] Imsland A. K. Reynolds P. Nytrø A. V. Eliassen G. Hangstad T. A. Jónsdóttir Ó. D. B. (2016b). Effects of lumpfish size on foraging behaviour and co-existence with sea lice infected Atlantic salmon in sea cages. *Aquaculture* 465 19–27. 10.1016/j.aquaculture.2016.08.015

[B45] Imsland A. K. Hanssen A. Nytrø A. V. Reynolds P. Jonassen T. M. Hangstad T. A. (2018a). It works! lumpfish can significantly lower sea lice infestation in large-scale salmon farming. *Biol. Open* 7:bio036301. 10.1242/bio.036301 30177547 PMC6176945

[B46] Imsland A. K. Reynolds P. Hangstad T. A. Jónsdóttir ÓD. B. Noble T. Wilson M. (2018b). Feeding behaviour and growth of lumpfish (*Cyclopterus lumpus* L.) fed with feed blocks. *Aquac. Res.* 49 2006–2012. 10.1111/are.13657

[B47] Imsland A. K. D. Reynolds P. Hangstad T. A. Kapari L. Maduna S. N. Hagen S. B. (2021). Quantification of grazing efficacy, growth and health score of different lumpfish (*Cyclopterus lumpus* L.) families: possible size and gender effects. *Aquaculture* 530:735925. 10.1016/j.aquaculture.2020.735925

[B48] Imsland A. K. D. Reynolds P. Jonassen T. M. Hangstad T. A. Adron J. Elvegård T. A. (2019). Comparison of diet composition, feeding, growth and health of lumpfish (*Cyclopterus lumpus* L.) fed either feed blocks or pelleted commercial feed. *Aquac. Res.* 50 1952–1963. 10.1111/are.14083

[B49] Imsland A. K. D. Reynolds P. Lorentzen M. Eilertsen R. A. Micallef G. Tvenning R. (2020). Improving survival and health of lumpfish (*Cyclopterus lumpus* L.) by the use of feed blocks and operational welfare indicators (OWIs) in commercial Atlantic salmon cages. *Aquaculture* 527:735476. 10.1016/j.aquaculture.2020.735476

[B50] Iversen M. Finstad B. McKinley R. S. Eliassen R. A. (2003). The efficacy of metomidate, clove oil, Aqui-S ™ and Benzoak ^®^ as anaesthetics in Atlantic salmon (*Salmo salar* L.) smolts, and their potential stress-reducing capacity. *Aquaculture* 221 549–566. 10.1016/s0044-8486(3)00111-x

[B51] Iversen M. Finstad B. Nilssen K. J. (1998). Recovery from loading and transport stress in Atlantic salmon (*Salmo salar* L.) smolts. *Aquaculture* 168 387–394. 10.1016/s0044-8486(98)00364-0

[B52] Iversen M. H. Eliassen R. A. (2014). The effect of allostatic load on hypothalamic–pituitary–interrenal (HPI) axis before and after secondary vaccination in Atlantic salmon postsmolts (*Salmo salar* L.). *Fish Physiol. Biochem.* 40 527–538. 10.1007/s10695-013-9863-x 24045864

[B53] Jørgensen E. H. Haatuft A. Puvanendran V. Mortensen A. (2017). Effects of reduced water exchange rate and oxygen saturation on growth and stress indicators of juvenile lumpfish (*Cyclopterus lumpus* L.) in aquaculture. *Aquaculture* 474 26–33. 10.1016/j.aquaculture.2017.03.019

[B54] Kilkenny C. Browne W. Cuthill I. C. Emerson M. Altman D. G. (2010). Animal research: reporting *in vivo* experiments: the ARRIVE guidelines. *Br. J. Pharmacol.* 160 1577–1579. 10.1111/j.1476-5381.2010.00872.x 20649561 PMC2936830

[B55] Lima S. L. Dill L. M. (1990). Behavioral decisions made under the risk of predation: a review and prospectus. *Can. J. Zool.* 68 619–640. 10.1139/z90-092

[B56] López-Patiño M. A. Skrzynska A. K. Naderi F. Mancera J. M. Míguez J. M. Martos-Sitcha J. A. (2021). High stocking density and food deprivation increase brain monoaminergic activity in gilthead sea bream (*Sparus aurata*). *Animals* 11:1503. 10.3390/ani11061503 34067338 PMC8224653

[B57] McEwen B. S. (1998). Stress, adaptation, and disease. allostasis and allostatic load. *Ann. N.Y. Acad. Sci.* 840 33–44. 10.1111/j.1749-6632.1998.tb09546.x 9629234

[B58] McEwen B. S. Wingfield J. C. (2003). The concept of allostasis in biology and biomedicine. *Horm. Behav.* 43 2–15. 10.1016/s0018-506x(02)00024-7 12614627

[B59] Messias J. P. M. Paula J. R. Grutter A. S. Bshary R. Soares M. C. (2016a). Dopamine disruption increases negotiation for cooperative interactions in a fish. *Sci. Rep.* 6:20817. 10.1038/srep20817 26853241 PMC4745044

[B60] Messias J. P. M. Santos T. P. Pinto M. Soares M. C. (2016b). Stimulation of dopamine D1 receptor improves learning capacity in cooperating cleaner fish. *Proc. R. Soc. B.* 283:20152272. 10.1098/rspb.2015.2272 26791613 PMC4795016

[B61] Mormède P. Andanson S. Aupérin B. Beerda B. Guémené D. Malmkvist J. (2007). Exploration of the hypothalamic-pituitary-adrenal function as a tool to evaluate animal welfare. *Physiol. Behav.* 92 317–339. 10.1016/j.physbeh.2006.12.003 17234221

[B62] Nilsson Sköld H. Aspengren S. Wallin M. (2013). Rapid color change in fish and amphibians - function, regulation, and emerging applications. *Pigment Cell Melanoma Res.* 26 29–38. 10.1111/pcmr.12040 23082932

[B63] North B. P. Turnbull J. F. Ellis T. Porter M. J. Migaud H. Bron J. (2006). The impact of stocking density on the welfare of rainbow trout (*Oncorhynchus mykiss*). *Aquaculture* 255 466–479. 10.1016/j.aquaculture.2006.01.004

[B64] Nytrø A. V. Vikingstad E. Foss A. Hangstad T. A. Reynolds P. Eliassen G. (2014). The effect of temperature and fish size on growth of juvenile lumpfish (*Cyclopterus lumpus* L.). *Aquaculture* 434 296–302. 10.1016/j.aquaculture.2014.07.028

[B65] Øverli Ø Nordgreen J. Mejdell C. M. Janczak A. M. Kittilsen S. Johansen I. B. (2014). Ectoparasitic sea lice (*Lepeophtheirus salmonis*) affect behavior and brain serotonergic activity in Atlantic salmon (*Salmo salar* L.): perspectives on animal welfare. *Physiol. Behav.* 132 44–50. 10.1016/j.physbeh.2014.04.031 24792663

[B66] Øverli Ø Pottinger T. G. Carrick T. R. Øverli E. Winberg S. (2001). Brain monoaminergic activity in rainbow trout selected for high and low stress responsiveness. *Brain Behav. Evol.* 57 214–224. 10.1159/000047238 11641559

[B67] Paula J. R. Messias J. P. Grutter A. S. Bshary R. Soares M. C. (2015). The role of serotonin in the modulation of cooperative behavior. *Behav. Ecol.* 26 1005–1012. 10.1093/beheco/arv039

[B68] Pottinger T. G. Carrick T. R. (2001). Stress responsiveness affects dominant-subordinate relationships in rainbow trout. *Horm. Behav.* 40 419–427. 10.1006/hbeh.2001.1707 11673915

[B69] Powell A. Treasurer J. W. Pooley C. L. Keay A. J. Lloyd R. Imsland A. K. (2017). Use of lumpfish for sea-lice control in salmon farming: challenges and opportunities. *Rev. Aquac.* 10:12194. 10.1111/raq.12194

[B70] R Development Core Team (2013). *R: A Language and Environment for Statistical Computing.* Available online at: https://www.r-project.org (accessed February 15, 2021)

[B71] Ramírez-Calero S. Paula J. R. Otjacques E. Rosa R. Ravasi T. Schunter C. (2021). *Neuro-Molecular Characterization of Fish Cleaning Interactions.* Available online at: https://www.biorxiv.org/content/10.1101/2021.06.22.449532v1 (accessed August 13, 2021) 10.1038/s41598-022-12363-6 PMC9119974 35589869

[B72] Ranjbar-Slamloo Y. Fazlali Z. (2020). Dopamine and noradrenaline in the brain; overlapping or dissociate functions? *Front. Mol. Neurosci.* 12:334. 10.3389/fnmol.2019.00334 32038164 PMC6986277

[B73] Rankin C. H. Abrams T. Barry R. J. Bhatnagar S. Clayton D. F. Colombo J. (2009). Habituation revisited: an updated and revised description of the behavioral characteristics of habituation. *Neurobiol. Learn. Mem.* 92 135–138. 10.1016/j.nlm.2008.09.012 18854219 PMC2754195

[B74] Rupia E. J. Binning S. A. Roche D. G. Lu W. (2016). Fight-flight or freeze-hide? Personality and metabolic phenotype mediate physiological defence responses in flatfish. *J. Anim. Ecol.* 85 927–937. 10.1111/1365-2656.12524 27044558

[B75] Schjolden J. Winberg S. (2007). Genetically determined variation in stress responsiveness in rainbow trout: behavior and neurobiology. *Brain Behav. Evol.* 70 227–238. 10.1159/000105486 17914254

[B76] Schreck C. B. (2010). Stress and fish reproduction: the roles of allostasis and hormesis. *Gen. Comp. Endocrinol.* 165 549–556. 10.1016/j.ygcen.2009.07.004 19596332

[B77] Selye H. (1975). “«Homeostasis and heterostasis»,” in *Trauma: Clinical and Biological Aspects*, ed. Day S. B. (Boston, MA: Springer), 25–29. 10.1007/978-1-4684-2145-3_2

[B78] Shapiro S. S. Wilk M. B. (1965). An analysis of variance test for normality (complete samples). *Biometrika* 52 591–611. 10.2307/2333709

[B79] Skår M. W. Haugland G. T. Powell M. D. Wergeland H. I. Samuelsen O. B. (2017). Development of anaesthetic protocols for lumpfish (*Cyclopterus lumpus* L.): effect of anaesthetic concentrations, sea water temperature and body weight. *PLoS One* 12:e0179344. 10.1371/journal.pone.0179344 28678815 PMC5497946

[B80] Soares M. C. (2017). The neurobiology of mutualistic behavior: the cleanerfish swims into the spotlight. *Front. Behav. Neurosci.* 11:191. 10.3389/fnbeh.2017.00191 29089876 PMC5651018

[B81] Soares M. C. Bshary R. Mendonça R. Grutter A. S. Oliveira R. F. (2012). Arginine vasotocin regulation of interspecific cooperative behaviour in a cleaner fish. *PLoS One* 7:e39583. 10.1371/journal.pone.0039583 22802939 PMC3389009

[B82] Soares M. C. Paula J. R. Bshary R. (2016). Serotonin blockade delays learning performance in a cooperative fish. *Anim. Cogn.* 19 1027–1030. 10.1007/s10071-016-0988-z 27107861

[B83] Soares M. C. Santos T. P. Messias J. P. M. (2017). Dopamine disruption increases cleanerfish cooperative investment in novel client partners. *R. Soc. Open Sci.* 4:160609. 10.1098/rsos.160609 28572985 PMC5451786

[B84] Staven F. R. Nordeide J. T. Gesto M. Andersen P. Patel D. M. Kristensen T. (2021). Behavioural and physiological responses of lumpfish (*Cyclopterus lumpus*) exposed to Atlantic salmon (*Salmo salar*) sensory cues. *Aquaculture* 544:737066. 10.1016/j.aquaculture.2021.737066

[B85] Staven F. R. Nordeide J. T. Imsland A. K. Andersen P. Iversen N. S. Kristensen T. (2019). Is habituation measurable in lumpfish *Cyclopterus lumpus* when used as cleaner fish in Atlantic salmon *Salmo salar* aquaculture? *Front. Vet. Sci.* 6:227. 10.3389/fvets.2019.00227 31338371 PMC6629887

[B86] Strand Å Alanärä A. Staffan F. Magnhagen C. (2007). Effects of tank colour and light intensity on feed intake, growth rate and energy expenditure of juvenile Eurasian perch, Perca fluviatilis L. *Aquaculture* 272 312–318. 10.1016/j.aquaculture.2007.08.052

[B87] Summers C. H. Winberg S. (2006). Interactions between the neural regulation of stress and aggression. *J. Exp. Biol.* 209 4581–4589. 10.1242/jeb.02565 17114393

[B88] Sunardi A. T. Manatunge J. Fujino T. (2007). The effects of predation risk and current velocity stress on growth, condition and swimming energetics of Japanese minnow (*Pseudorasbora parva*). *Ecol. Res.* 22 32–40. 10.1007/s11284-006-0186-6

[B89] Swallow J. G. Bubak A. N. Grace J. L. Editors G. (2016). The role of monoamines in modulating behavior. *Cur. Zool.* 62 253–255. 10.1093/cz/zow046 29491912 PMC5804270

[B90] Teles M. C. Dahlbom S. J. Winberg S. Oliveira R. F. (2013). Social modulation of brain monoamine levels in zebrafish. *Behav. Brain Res.* 253 17–24. 10.1016/j.bbr.2013.07.012 23850359

[B91] Toms C. N. Echevarria D. J. Jouandot D. J. (2010). A methodological review of personality-related studies in fish: focus on the shy-bold axis of behavior. *Int. J. Comput. Psychol.* 23 1–25.

[B92] Torrissen O. Jones S. Asche F. Guttormsen A. Skilbrei O. T. Nilsen F. (2013). Salmon lice - impact on wild salmonids and salmon aquaculture. *J. Fish Dis.* 36 171–194. 10.1111/jfd.12061 23311858 PMC3675643

[B93] Vindas M. A. Fokos S. Pavlidis M. Höglund E. Dionysopoulou S. Ebbesson L. O. E. (2018). Early life stress induces long-term changes in limbic areas of a teleost fish: the role of catecholamine systems in stress coping. *Sci. Rep.* 8:5638. 10.1038/s41598-018-23950-x 29618742 PMC5884775

[B94] White J. R. Meekan M. G. McCormick M. I. Ferrari M. C. O. (2013). A comparison of measures of boldness and their relationships to survival in young fish. *PLoS One* 8:e68900. 10.1371/journal.pone.0068900 23874804 PMC3712919

[B95] Whittaker B. A. Consuegra S. de Leaniz C. G. (2021). *Personality Profiling May Help Select Better Cleaner Fish for Sea-Lice Control In Salmon Farming.* Available online at: https://www.biorxiv.org/content/10.1101/2021.05.21.444956v1 (accessed August 15, 2021)

[B96] Winberg S. Nilsson A. Hylland P. Söderstöm V. Nilsson G. E. (1997). Serotonin as a regulator of hypothalamic-pituitary-interrenal activity in teleost fish. *Neurosci. Lett.* 230 113–116. 10.1016/S0304-3940(97)00488-6 9259477

[B97] Winberg S. Thörnqvist P.-O. (2016). Role of brain serotonin in modulating fish behavior. *Cur Zool.* 62 317–323. 10.1093/cz/zow037 29491919 PMC5804243

[B98] Wingfield J. C. (2005). The concept of allostasis: coping with a capricious environment. *J. Mammal.* 86 248–254. 10.1644/bhe-004.1

[B99] Yuan M. Chen Y. Huang Y. Lu W. (2018). Behavioral and metabolic phenotype indicate personality in zebrafish (Danio rerio). *Front. Physiol.* 9:653. 10.3389/fphys.2018.00653 29899710 PMC5988878

